#  The application of artificial intelligence-generated content in ophthalmology education

**DOI:** 10.3389/fmed.2025.1617537

**Published:** 2025-07-18

**Authors:** Yinzongxiao Wang, Yun Zhao, Jia Li

**Affiliations:** Department of Ophthalmology, The Second Hospital of Jilin University, Changchun, China

**Keywords:** ophthalmology education, AI, AIGC, generative adversarial networks, deep learning, digitization

## Abstract

With the rise of generative artificial intelligence (AI) technology, AI has played a significant role in ophthalmology clinical applications, and AI-generated content (AIGC) has shown great potential in ophthalmology education. Specifically, AIGC plays an important role in lesson plan generation, simulated cases, and disease diagnosis, but its application also faces challenges related to the invasion of patient privacy and the accuracy of generated content. To better enable AIGC and promote the development of ophthalmology education, this article provides an overview of AI and ophthalmology and the application, challenges, and development prospects of AIGC in ophthalmology education. References for related research as well as practice are also provided.

## 1 Introduction

Artificial intelligence-generated content (AIGC) is a specific application of AI technology in the field of content generation. With the in-depth study of AIGC technology in ophthalmology teaching, on the one hand, AIGC technology can generate teaching materials for ophthalmology teaching, and provide ophthalmology students with a platform for interactive learning and simulation training to improve their practical skills. On the other hand, it also faces many challenges, such as medical accuracy and ethical issues. AIGC technology can generate teaching materials for ophthalmology education and provide a platform for ophthalmology students to improve their practical skills through interactive learning and simulation training. However, it also faces many challenges, including the medical accuracy of the generated content and ethical issues.

## 2 Application of AIGC in ophthalmology clinics

As the global population continues to age, medical costs have increased. The current doctor–patient ratio in China is 1:950 (1), which is much lower than the international level. Ophthalmologists in China are often face a heavy load of clinical tasks and have limited time to engage in academic research. Senior physicians often do not have sufficient time to supervise the training of medical students due to their clinical obligations. In addition, the COVID-19 pandemic has reduced training opportunities for interns (2). All of the above factors pose a challenge to high-quality training for ophthalmologists. However, AI can leverage arithmetic power to analyze ocular information and process data at the pixel level that cannot be recognized by the naked eye, thereby increasing the speed of diagnosis (3). This can reduce the amount of clinical work performed by doctors and enable them to devote more time and energy to educating young doctors. In addition, to address these challenges, the Lancet Commission has made a new proposal to move away from traditional apprenticeship and time-based medical training programs to a more competency-based curriculum (4). This proposal emphasizes the introduction of new concepts in team-based care and patient-centered approaches, as well as the integration of new technologies [e.g., artificial intelligence (AI) and simulation] into the training curriculum (5). It is well known that ophthalmology has been at the forefront of AI development and application in clinical settings, including in the areas of detection and classification of diseases such as diabetic retinopathy, age-related macular degeneration (AMD), primary glaucoma, and retinopathy of prematurity (ROP) (6). For example, Wang et al. (7) proposed the automatic diagnosis of diabetic fundus lesions based on Region-based Fully Convolutional Networks (R-FCN) algorithm, and the optimized R-FCN algorithm recognition model can accurately complete the grading of lesion degree and localize the lesion area. Chou et al. (8) used nearly 700 fundus photographs to validate their deep learning (DL) model to diagnose AMD, in addition to disease diagnosis, the AI model can be used to predict the disease severity and progression of AMD patients. Diaz-Pinto et al. (9) built a retinal image compositor and semi-supervised classifier using generative adversarial networks (GANs) technology for glaucoma detection, the image compositor was able to generate realistic images with glaucomatous. The image compositor can generate realistic images with glaucomatous features. The classifier can distinguish between glaucomatous and normal images with high accuracy, and many glaucomatous retinal images can be generated by using the image compositor and the classifier. The application of AI in the field of ROP is mainly to detect and grade ROP, and the i-ROP-DL system developed based on a convolutional neural network (CNN) can improve the objectivity of ROP diagnosis and accessibility of screening (10).

Artificial intelligence has also shown great potential in ophthalmology, including in screening, diagnosing, and grading diseases, detecting disease recurrence, quantifying treatment efficacy, and identifying new diagnostic and therapeutic approaches (11). Among them, AIGC is an important branch in the field of AI, and AIGC refers to content generated by AI algorithms. The China Academy of Information and Communications Technology (CAICT) and the Jingdong Discovery Institute (JDDI) jointly released the *Artificial Intelligence-Generated Content (AIGC) Whitepaper* in September 2022 (12) and defined AIGC as “a class of content classified from the perspective of content producers, a method of content production, and a collection of technologies used for the automated generation of content.” Through technologies such as GANs and DL models, AI systems are able to learn and generate content, including text, images, video, audio, and other content. Overall, AIGC is summarized as a new type of generative web information content that accompanies the evolution of network morphology and changes in AI technology (13).

The GAN technique involves a generative model that generates images using the training dataset and a discriminative model that determines whether the images are from the real training dataset rather than generated. The generative model and discriminative model are constantly competing with each other and improving each other to produce a synthetic image that is almost indistinguishable from the real image (14). By using the GAN technique a large number of random and diverse medical images can be synthesized and can be used to generate training datasets for CNN in the future (15, 16). Yuhao et al. (17) augmented the image data of neonatal retinal hemorrhage by constructing a GAN structure for paired data and proposed a new grading criterion for quantifying neonatal retinal hemorrhage, which can measure the relative position of hemorrhage points to the macular danger zones. This makes the grading results more accurate, which can greatly reduce the diagnostic workload of doctors and improve student outcomes in fundus imaging learning programs.

**FIGURE 1:**
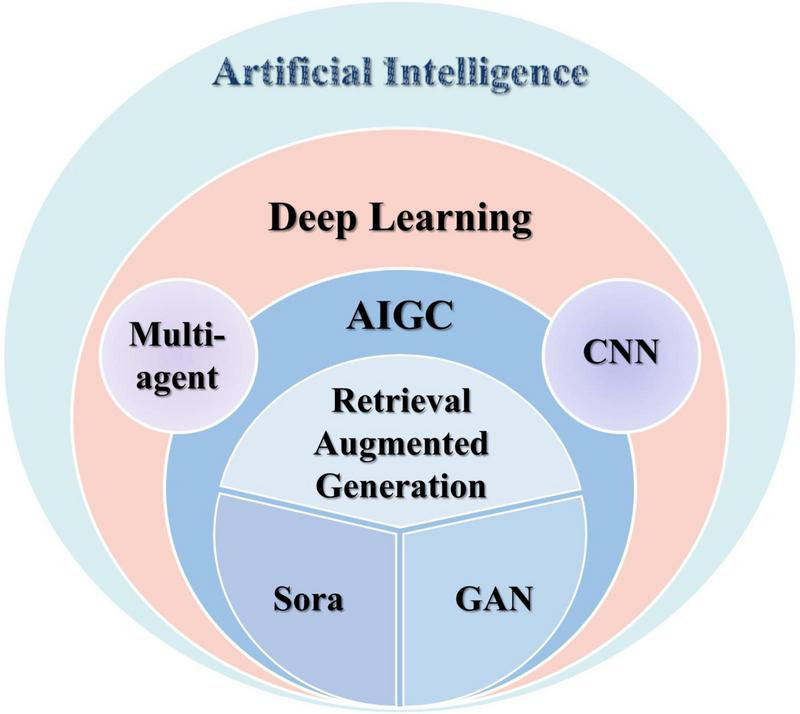
This figure shows the relationship between the various AI technologies mentioned in this article. AIGC: artificial intelligence-generated content is a specific application of AI technology in the field of content generation. CNN: convolutional neural network, a deep learning model designed for efficient feature extraction from structured data, especially images. GAN: Generative adversarial network, a deep learning framework consisting of a generator and a discriminator trained in opposition to synthesize realistic data. Sora: spatiotemporal object-centric rendering architecture, a text-to-video diffusion model by OpenAI that is capable of generating high-fidelity, temporally consistent videos from textual prompts.

The popularity of DL techniques in the field of ophthalmology based on image-based diagnostic systems is gradually increasing (18). De Fauw et al. (19) trained a DL framework using three-dimensional optical coherence tomography (OCT) images to enable the detection of not only one disease but even more than 50 common retinal diseases. Automatic diagnosis and assisted diagnosis of ophthalmic diseases can be achieved by training DL models that use a large amount of ophthalmic image data for learning and recognition. DL models are also able to extract features from complex ophthalmic images and perform accurate classification and diagnosis (20). For example, cataracts can be classified into nuclear cataracts (NCs), cortical cataracts (CCs), and posterior subcapsular cataracts (PSCs) on the basis of the cloudy part of the lens. Keenan et al. (21) developed the 2022 DL model DeepLensNet for automatic diagnosis and quantitative classification of these three types of cataracts, with a mean MSE of 0.23 in the classification of NCs, which was significantly better than that of 14 ophthalmologists (mean MSE of 0.98) and 24 medical students (mean MSE of 1.24). The study provides an efficient tool for the study of senile cataracts. Since ophthalmological diagnosis relies heavily on imaging examinations, AI based on DL methods can rapidly process image information and improve the efficiency of ophthalmologists. The application of AI in ophthalmology will hopefully solve the problem of a large population base and shortage of medical resources in China (22). Because AI technology in ophthalmic medicine has the characteristics of multimodal imaging modality, high-resolution image quality, low cost, and non-invasive, which is very suitable for ophthalmic medicine, and diagnostic imaging is the main goal of DL, we should give full play to AI in ophthalmic medicine (23). In addition, in ophthalmology clinical work, AIGC is able to automatically obtain abnormal information in fundus pictures through CNN training and give corresponding diagnoses, which is expected to be an effective tool for primary care screening (24). (The relationship of various techniques is shown in Figure 1).

At present, AI technology is developing rapidly. In 2018, the United States approved the first AI device for detecting diabetic retinopathy to provide diagnostic decision-making (25), and China approved the first batch of AI software for diagnosing diabetic retinopathy in 2020. They are the innovative product “fundus image assisted diagnosis software for diabetic retinopathy” produced by Shenzhen Silicon-based Intelligent Technology Co., LTD and the innovative product “fundus image assisted diagnosis software for diabetic retinopathy” produced by Shanghai Yingjia Medical Technology Co., LTD (26). In the future, AI will have a greater application prospect in the field of ophthalmology, so we should attach importance to the application of AIGC in the field of ophthalmology and realize the innovation of ophthalmology teaching technology (27).

The use of AI includes the use of various models, each of which plays distinct roles in ophthalmic medical education and clinical practice. In research, clearly distinguishing between discriminative models and generative models will facilitate a deeper exploration of their functions in ophthalmology education. Although both discriminative and generative AI models are crucial in ophthalmology education, only generative models fall under the category of AIGC. For example, GANs can generate high-quality synthetic images for the segmentation of endoscopic surgical tools (28), which are useful for case teaching; large language models (LLMs) can be employed to create clinical teaching dialogs, simulate doctor–patient interactions, or provide personalized explanations for students. Discriminative models, such as CNNs, are mainly used for image classification and do not directly generate images. For example, training a CNN to recognize the grade of DR in fundus images (29) can provide students with automated diagnostic feedback, increasing their diagnostic skills. Table 1 provides a brief comparison of the application characteristics of the two types of models in ophthalmology education.

**TABLE 1:** Comparison of the applications of discriminative and generative artificial intelligence models in ophthalmology education.

AI models	Discriminative models	Generative models
Application	Image classification/diagnosis (30)	Synthetic pathological images (31)
	Lesion detection and grading (32)	Generated educational dialog/text (33)
	No content generation	Capable of content generation

## 3 AIGC and ophthalmology teaching

**FIGURE 2:**
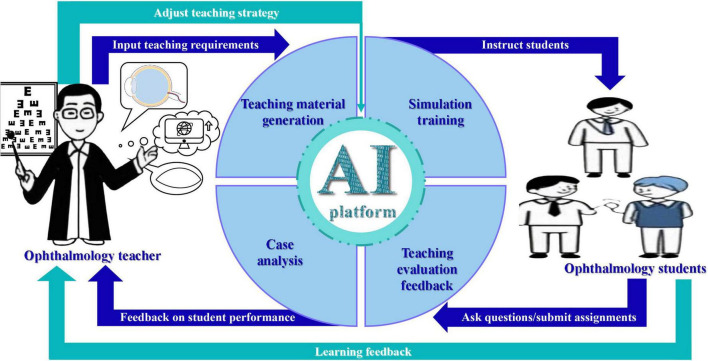
This figure shows the current teaching process of ophthalmology teachers and ophthalmology students using the AIGC platform.

Ophthalmology teaching in China includes the undergraduate education stage, the postgraduate education stage, the residency training stage, and the continuing medical education stage. Ophthalmology teaching at the undergraduate level is dominated by theoretical courses, with a relatively low proportion of practical courses, and too much emphasis is placed on the dominant position of teachers in teaching, neglecting the cultivation of students’ active learning (34). As China’s high-quality resources are concentrated in large cities and key institutions, the quality of postgraduate teaching varies among regions. Because of the lack of national standards, there are significant regional differences in ophthalmology residency training in China compared with the United States, and the quality of training varies (35). Therefore, we need to focus on the combination of theory and practice in the teaching process, establish a national unified teaching quality supervision system, and promote virtual reality (VR) and augmented reality (AR) technology, so that students can complete complex operation training in the virtual environment. AIGC has the potential to transform the current educational model by leveraging big data, cloud computing, and other integrated educational resources. These technologies can be effectively applied to ophthalmology education and talent training. In the context of AI, the teaching model is centered primarily around teacher–AI–student interactions (36) (Figure 2). The use of multi-subject interaction dialogue teaching and diversified teaching scenarios promotes the personalized development of teaching and the cultivation of innovative talents (37). The current applications of AIGC in ophthalmology teaching are as follows:

### 3.1 Generation of ophthalmic knowledge content

#### 3.1.1 Generation of teaching materials

Artificial intelligence-generated content can generate teaching materials, handouts, and case study materials by combining ophthalmology teaching requirements with its automatic generation capability (38–40). Burlina et al. (41) demonstrated that GANs can be employed to generate color retinal images representing various stages of AMD. Furthermore, a deep learning system (DLS), trained exclusively on these synthetic images, was able to accurately differentiate between referable and non-referable AMD when evaluated on real-world data. DLS was able to distinguish well between non-referential AMD and referential AMD when tested against real images, indicating that synthetic ophthalmic images can train DL algorithms while being able to increase the size of the training sample (42). Generating images by GAN can solve the problem of data shortage and can be used for ophthalmology teaching (43). GAN has a greater advantage in ophthalmology auxiliary diagnosis problems, such as retinal blood vessel segmentation and synthesizing pictures of AMD (41). Zheng et al. (44) synthesized OCT images using GAN and found that GAN-synthesized images can be used for neural network algorithm research, and can also be used as clinical education sample images. Long et al. (45) developed an AI-assisted teaching module for congenital cataracts based on the CC-cruiser platform, which can deepen students’ understanding of the disease. In addition, if more real cataract preoperative and postoperative images are used to optimally train the GANs model, it will improve its practical value and better apply it to clinical data and clinical teaching (46). To better demonstrate the AI-generated ophthalmic teaching materials, Table 2 is presented below:

**TABLE 2:** Artificial intelligence-generated content generates a table of ophthalmology teaching materials.

AI technology	Teaching material content	Application scenario
GAN	Create a composite OCT image	Macular disease image diagnosis education (47)
GAN	High-resolution optical coherence tomography (AS-OCT) images of the anterior segment (48)	High definition composite AS-OCT images
GAN	Synthesize ultra-wide angle (UWF) retinal images	Using composite images to teach students how to identify disease in UWF retinal images (49)
DCNN	MRI image data for detecting thyroid-associated ophthalmopathy (TAO) (50)	Obtain these features directly from the MRI of the eye socket to assist in the clinical staging of TAO patients
Deep learning	Diabetic retinopathy (DR) and diabetic macular edema (DMO) in retinal fundus photographs were automatically detected and labeled (51)	The annotated image materials can be used by teachers to explain the characteristics of the lesions and help students understand the appearance and location of the lesions more intuitively

#### 3.1.2 Personalized generation for students

Compared with traditional ophthalmology teaching methods such as bedside instruction—which may cause patient discomfort or resistance and thus compromise teaching quality—the integration of AI-assisted teaching with conventional demonstration methods can increase patient cooperation. Moreover, by utilizing AI systems to develop personalized learning plans for each student, this approach can improve individual learning strategies (52) and ultimately increase the overall quality of ophthalmology education (53).

### 3.2 Interactive analysis and simulation training

#### 3.2.1 Interactive analysis

Artificial intelligence-generated content can provide students with opportunities for self-directed learning and enhance adaptability as well as interactivity in digital environments (54). Generative AI facilitates ophthalmic clinical trials and research by simulating trial parameters, processing large-scale data, and optimizing treatment regimens. Generative AI can be used to enhance student learning experiences and outcomes by creating interactive and adaptive learning environments for ophthalmic student training (55). In addition to current diagnostic and predictive tasks, AI methods can be used to provide ophthalmologists with additional information that cannot be obtained through visual inspection alone. For example, objective quantification of corneal ulcer areas in combination with segmentation and detection techniques could help ophthalmologists to accurately assess treatment efficacy during follow-up visits. When the ulcer area decreases, it indicates improvement; conversely, if the area increases, it suggests that the condition may be worsening and the treatment strategy needs to be reassessed (56). AIGC can be used not only to provide assistance to ophthalmologists but also to train ophthalmology medical students. Students can discuss and learn by combining cases with the results of AI analysis, and can more intuitively understand the changes in the condition and the effects of treatment, improving their clinical practice while gaining a deeper understanding of the disease development process as well as treatment options (56). Most of the traditional ophthalmology teaching cases only show the performance of a certain patient and a certain disease at a certain stage of development, it is difficult to show the complete development of the disease from the early stage to the end stage. Through AIGC technology, the developmental trajectory of the disease from the onset to the end stage can be simulated dynamically, and combined with the current clinical treatment interventions, the regression of the disease under different intervention strategies can be shown intuitively. This dynamic and comprehensive display effect cannot be achieved by traditional clinical teaching. In actual clinical practice, patients often receive therapeutic interventions during disease development, coupled with patient compliance and continuity of care, it is often difficult to completely record the natural progression of the disease without intervention. Therefore, AIGC technology has significant advantages in ophthalmology teaching.

#### 3.2.2 Simulation training

Artificial intelligence can explain complex medical concepts in simple terms, thereby helping students learn, and AI can provide case-based scenario exercises for testing students’ knowledge mastery. However, the training content should be reviewed by ophthalmologists before use (52). GANs can generate a diverse set of imaging training datasets by combining simple background images with a limited number of surgical images (28) that are used to create a variety of simulations and practice scenarios. A multi-institutional collaboration between King’s College and Imperial College in London developed a virtual reality (VR) surgical anatomy course for robot-assisted radical prostate cancer surgery (57), which could also be used to train ophthalmologists by simulating different surgical conditions to provide ophthalmologists with a more realistic and immersive learning experience. In addition, with the continuous development of AIGC, various cutting-edge technologies have emerged, including Retrieval Augmented Generation (RAG), Multi-Agent, and Sora. Multi-agent technology can simulate a real medical environment through the collaboration and communication between intelligence (58). For example, an ophthalmology glaucoma surgery scenario can be established via multi-agent technology, which includes multiple intelligences, such as surgeons, nurses, anesthesiologists, and simulated patients. To formulate a reasonable surgical plan, students need to interact with AIs in ways that are appropriate for their knowledge level and in ways that take into account changes in the patient’s condition. The students need to interact with each intelligent body in this environment according to their knowledge and changes in the patient’s condition to develop a reasonable surgical plan, and this teaching mode can greatly improve the students’ clinical practice ability. The video generation technology represented by Sora can generate three-dimensional animation videos of various structures of the eye (58), which can deepen the student’s understanding of the anatomical structures of the eye compared with the traditional teaching materials. In addition, Sora technology can also generate error simulations, such as simulating improper operations during surgery, which can be used to test students’ knowledge, thus deepening their impression and avoiding mistakes in practice. AIGC-driven virtual patient interaction and clinical scenario simulation can provide immersive learning for medical students, thus improving students’ knowledge transfer and skill application ability (59).

### 3.3 Teaching evaluation and feedback

Generative adversarial networks can autonomously generate images of various fundus diseases during training. Once validated by senior ophthalmologists, these images can be uploaded to educational platforms for use in ophthalmology training, particularly to support the education of primary care physicians. Also, GAN can generate various fundus images for student assessment and can protect patient privacy (60). In addition, Kim et al. (61) reported that a DLS can classify the level of user expertise and can provide a rating of the surgeon’s ability to perform a capsulotomy in cataract surgery. The data collected with AI can be used to assess students and monitor their surgical skills to prevent adverse outcomes (62).

## 4 Challenges of AIGC in teaching ophthalmology

### 4.1 Technical challenges

#### 4.1.1 Training dataset

The quality of an AIGC model depends heavily on the quality of the training data (63), which means that if the training dataset of AIGC contains false and harmful information, it will affect the quality of the content output by AIGC (64). And the results produced by AI are often affected by the size of its training set, for example, for some rare diseases, AI may not be able to produce accurate results. The current datasets at home and abroad lack standardization, and their interpretive reliability varies according to the standards of their respective teams, so the establishment of standard datasets will promote the development of AI in China (22). Although GAN-synthesized images can show many pathological features, they may also contain false information and need to be screened when used (43). In addition, most algorithms are developed using images acquired from medical imaging devices and rely on expert-based, subjective classification and labeling. However, owing to the inherent variability in expert interpretations (65), such subjectively labeled data may omit clinically significant features, potentially leading to misclassification. This, in turn, can negatively affect the quality of the training dataset and hinder both ophthalmic diagnosis and educational outcomes. Therefore, to better apply AI, we need to further establish standardized, high-quality databases covering all areas of ophthalmology, while AI-based analysis of multimodal data can be strengthened to improve diagnostic accuracy and clinical teaching value (27).

#### 4.1.2 Black box problem

The black box problem refers to the fact that in some cases, we can observe the inputs and outputs of the system, but we cannot understand the specific working principle or logic inside the system. In the fields of machine learning and AI, the “black-box” problem typically refers to the difficulty in interpreting the internal mechanisms of complex models such as deep neural networks (66). In other words, AI systems are often unable to clearly demonstrate their decision-making processes (67), which necessitates that physicians remain ultimately responsible for clinical decisions. The inherent opacity of such systems may undermine physicians’ trust in AI-assisted diagnostics (68).

#### 4.1.3 Deep learning algorithms

Noisy data in DL’s training sets also affects the performance of their predictive models (69), and some training sets ignore overall patient eye covariates (70), visual field, race, etc. Although DL algorithms have demonstrated superior accuracy and efficiency compared with those of humans in certain tasks—such as classifying a limited number of predefined diseases, assessing disease severity, performing image structure segmentation, and predicting disease prognosis—they remain limited in their ability to diagnose previously unseen or untrained conditions. Moreover, unlike human physicians, these algorithms are unable to interact with patients and therefore cannot engage in diagnostic reasoning or therapeutic decision-making in a clinical context (71).

#### 4.1.4 ChatGPT

Large language model (LLM) is a kind of AIGC model, the typical LLM model is ChatGPT, which can be used for text generation, dialogue system, and translation. However, false information and illogical reasoning generated by AI can mislead humans and thus negatively influence clinical decisions. For example, in the treatment of neovascular glaucoma, which cannot be controlled by medication, ChatGPT incorrectly chose trabeculectomy instead of the use of an atrial fluid drainage device (72). In addition, ChatGPT may produce incorrect answers because it lacks real ophthalmic expertise and does not have the clinical experience of a professional ophthalmic practitioner (73). Although the ChatGPT can retrieve ophthalmic information from databases, it is unable to integrate to form new knowledge (74) and if the ChatGPT is exposed to misinformation during training, this may lead to its continued dissemination of incorrect information (75).

### 4.2 Ethical and regulatory issues

The ethical challenges faced by AI, when implemented in medicine, have been categorized into six main groups, including machine training ethics, machine accuracy ethics, patient-related ethics, physician-related ethics, sharing ethics, and the role of regulators (76). The development of AI needs to be accompanied by advanced data protection measures because training DLSs requires a large amount of medical data. However, these data often involve patient privacy; for example, DL algorithms can infer a patient’s age, sex, and even ethnicity from retinal photographs (70, 77). In ophthalmology-specific standardized exams, LLM performs poorly compared to humans with poor accuracy levels (78), so there have been concerns about inaccurate answers or explanations given by AI and issues such as academic integrity. In addition based on computer learning and AI algorithms that may be characterized by bias or favoritism toward certain groups, ophthalmic surgical training facilities are involved in issues such as data privacy, transparency, bias, accountability, and responsibility (79).

### 4.3 Challenges in practical applications

#### 4.3.1 Difficulty of successful algorithm development and successful application

Google’s DL-based diagnostic algorithm for the fundus glycoconjugate network, published in 2016, achieved performance comparable to that of expert ophthalmologists under ideal laboratory conditions. However, its effectiveness declined significantly in real-world settings because of factors such as low patient cooperation and suboptimal network infrastructure. This disparity highlights the substantial gap between successful algorithm development and practical clinical implementation (80). Significant gaps remain in the standardization of AI integration into clinical practice. These include the limited adoption of imaging standards, the lack of defined use cases for AI-based decision support systems, the absence of a unified data model to coordinate large-scale data repositories, and the lack of standardized protocols for interfaces and algorithmic outputs (81). Additionally due to the differences between patient populations, imaging devices, and scan types, some DL algorithms may still produce inaccurate results when applied to other external datasets despite the algorithms demonstrating high accuracy during development, making these algorithms of limited real-world applicability (82).

#### 4.3.2 Problems of co-operation between parties

Hospitals have a large amount of clinical data, but it is usually not well organized and they are not well connected to AI experts, thus preventing meaningful exploitation of a large amount of clinical data. Meanwhile, computer companies have a large number of AI experts but do not have access to clinical data (83). AI model sensitivity is affected by many aspects, such as the degree of patient cooperation and the level of physician operation (20). Meanwhile, in practical applications, if students rely too much on AI it will lead to the degradation of learning skills and the regression of personal cognitive ability (84).

To deal with the above challenges, we should promote the establishment of standardized, high-quality datasets, ensure the transparency of the source of AIGC training data for the black-box problem, and use visualization techniques to improve the interpretability of the model (58). In the face of false information generated by AIGC, corresponding laws and regulations should be improved (85). Real-time monitoring and auditing of generated content should be carried out, and false information should be blocked in a timely manner. In terms of ethics, corresponding guidelines should be formulated to protect patient privacy and avoid prejudice discrimination. Therefore successful use of AIGC in ophthalmology teaching requires the active participation of patients, ophthalmologists, supervisors, technicians, etc., as well as ensuring the protection of patient privacy, and jointly promoting the development of AIGC in ophthalmology teaching (71).

#### 4.3.3 Limitations of AIGC in multilingual medical education contexts

Ophthalmic medical education is essentially a field where multiple languages and cultures are interwoven. Although the application of AIGC in ophthalmology education is becoming increasingly widespread, most existing methods are still limited to a single language, usually English. When LLMs (such as ChatGPT) are used in non-English environments, their performance often varies significantly. For example, research by Panthie et al. (86) indicated that ChatGPT achieved a 91% pass rate in the EBO exam. However, there are still deficiencies in some specific fields. For example, in parasitological examinations, the performance of ChatGPT is not as good as that of well-trained medical students (87). Similarly, in Sakai et al.’s (88) research, the test results revealed that in a Japanese environment, the accuracy of the ChatGPT declined for questions with high translation dependence and those involving culturally specific terms. Furthermore, Sabaner et al.’s (89) research compared the performance of the ChatGPT-4 Omni and Gemini 1.5 Pro in the Swedish medical language proficiency test and reported that there were significant differences in their responses to questions in English and questions in Swedish. The current performance of AIGC in ophthalmic education applications in different language environments is still unstable, which may limit the fairness of accessing high-quality ophthalmic AI educational resources worldwide. This suggests that we should enhance cross-language verification and location-related adaptations to achieve the full integration of AIGC in medical education.

## 5 Prospects for the development of AIGC in ophthalmology teaching and learning

### 5.1 Prospects for the development of the technical aspects of AIGC in ophthalmology

#### 5.1.1 AI and OCT

Using DL algorithms for finer grading of sample features will be the future direction of research (90). Key methods for ophthalmic disease monitoring include OCT, and the use of DL methods for interpreting OCT images can improve the accuracy as well as the efficiency of OCT-based diagnosis (6). Integrating AI with OCT imaging has great potential to improve disease diagnostic accuracy and will play an important role in helping to improve patient prognosis as well as teaching ophthalmology (91).

#### 5.1.2 Migration learning techniques

In AIGC, migration learning can help the model utilize existing knowledge and experience when dealing with the task of fundus image classification to improve classification accuracy and efficiency. Data enhancement strategy refers to generating more training samples by transforming, expanding, or adding noise to the original data, thus improving the generalization ability and robustness of the model. For example, owing to the limited availability of pediatric ophthalmology data and the distinct clinical presentation of pediatric ophthalmic diseases, directly applying machine learning models trained on adult data to pediatric populations may result in a high error rate (43). However, transfer learning techniques (92) can help mitigate this issue by adapting adult-trained models to pediatric contexts, thereby compensating for data scarcity and supporting the training of medical students in pediatric ophthalmology.

#### 5.1.3 Establishment of databases

Establishing freely accessible, disease-specific or device-specific shared data resources would be highly beneficial for enabling computer scientists to evaluate and refine algorithmic designs. For example, the publicly available ImageNet dataset has played a pivotal role in numerous breakthroughs in image recognition. In the medical domain, the Kaggle organization’s publicly released dataset on DR has been widely adopted by algorithm developers. Similarly, the UK Biobank has provided open-access datasets for eye disease classification and predictive modeling, serving as a successful example of large-scale data sharing (83).

#### 5.1.4 Continuous optimization of artificial intelligence in ophthalmology

The performance of AI systems is often determined at the time of development, based mainly on solid data sets and static environments; however, the world is not static, and AI systems should be based on continuous learning techniques to learn new skills without forgetting previous solutions, and lifelong learning like clinicians (93). With the continuous development of technology, AI will also have a lot of room for development in areas such as assisting retinal teaching (94). In the future, we need to invest more efforts to gradually improve the process of collecting research samples for ophthalmic AI models as well as the corresponding guidelines and so on (20).

### 5.2 AIGC innovations in ophthalmology teaching models

#### 5.2.1 Development of digital learning curriculum

Medical education should adapt to the context of the times and actively explore the application of AI in ophthalmology teaching. Predictable applications for ophthalmology education should be enhanced, including generating active learning questions, simulating real cases, and providing answer explanations for standardized medical examinations (78). Medical schools should offer AI ethics courses that use advanced technologies to assist teaching and research to develop digital learning-related curricula (95). To better integrate AI with ophthalmic education, ophthalmic educators should develop longitudinal curricula for ophthalmic training that integrate AI, big data, informatics, and telemedicine (96). Telemedicine can be used not only to address patient access in remote areas but also to provide educational resources for ophthalmologists. We can develop and implement telemedicine projects based on AI technology and develop relevant curricula for ophthalmology medical students (97). Informatics, statistics, and computer courses are also needed as part of the pedagogical training to train future ophthalmologists driven by AI (98). In addition, to better adapt to the development of AI in ophthalmology, the teaching of future ophthalmologists should place more emphasis on the cultivation of humanistic elements such as professionalism and empathy.

#### 5.2.2 Incorporating telemedicine

Telemedicine uses electronic communication technology to deliver healthcare over long distances or over long periods (99), and its use in neovascular AMD and glaucoma has demonstrated that telemedicine can be a great convenience for patients (100). Distance education, such as the Global Education Network for Retinopathy of Prematurity (GEN-ROP), experiments conducted among ophthalmology students in the United States and Canada have shown significant improvements in the diagnostic accuracy of the students after participating in this program (101). The spread of AI will greatly improve the uneven distribution of healthcare resources, and AIGC can be fully utilized in ophthalmology learning in areas where specialists are scarce. Improving the diagnostic level of doctors can also reduce the cost of patient care, and medical students are also able to use AI to deal with complicated data when conducting scientific research (102).

#### 5.2.3 Incorporating virtual reality technology

The Eyesi Surgical Platform (VRMagic) is a VR ophthalmic surgical simulation platform that helps ophthalmologists and medical students train and practice surgical skills. Using VR technology, the platform provides a highly realistic ophthalmic surgical simulation environment that enables doctors to practice ophthalmic surgical techniques such as cataract surgery and vitrectomy in simulated scenarios, which can improve their skill levels and surgical performance. With the Eyesi Surgical Platform, doctors can practice repeatedly in a safe virtual environment to enhance their surgical skills, reduce surgical risks, and improve patient outcomes (103). In addition, VR headsets are increasingly being used in ophthalmology teaching. Through the use of VR headsets, students and surgeons can participate in live or recorded surgeries (104) in VR, including the surgical site, surgeon, and surgical environment, while performing virtual surgical operations and practices. This technology can provide a more intuitive, interactive, and immersive learning experience to help medical students better understand the complexities and details of ophthalmic surgery and improve their surgical skill levels. VR headsets can also be used for distance learning and training, allowing students and physicians to receive high-quality ophthalmology education at any time, from anywhere. Overall, the use of VR headsets in ophthalmology teaching has opened up a whole new world of possibilities and opportunities for medical education.

### 5.3 Chatbots in ophthalmology education

#### 5.3.1 Advantages and limitations of chatbots in ophthalmology education

In recent years, chatbots based on LLMs have gradually become important auxiliary tools in ophthalmic education. By automatically generating diverse clinical cases, this type of tool can provide immediate feedback when students are training, helping them identify and correct mistakes to improve their practical ability; additionally, the use of this tool can significantly reduce teaching costs. Moreover, this type of chatbot increases the accessibility of teaching content, especially in resource-constrained areas. Chatbots can offer relatively fair learning opportunities for ophthalmologists in various regions and effectively address the issue of insufficient teaching staff. They are also increasingly used in ophthalmology-related research, especially in examinations that involve multiple-choice questions (MCQs) (105). However, the wide application of this technology has also exposed many limitations. The accuracy of the generated content cannot be fully guaranteed, and there is a risk of “hallucinations,” that is, outputting fictional or inaccurate information (73), which is likely to mislead novice ophthalmology medical students. Furthermore, ophthalmology education is highly dependent on various image materials, whereas chatbots are relatively weak in regard to integrated visual information. Furthermore, overreliance on chatbots weakens students’ ability to think and judge independently (84). Sabaner et al. (105) noted that although chatbots have broad prospects in medical education, both their image-processing capabilities and ethical issues need to be further addressed. Moreover, the answers given by chatbots may vary due to the training data used and the chatbot version, which leads to problems with the reliability of the content that chatbots generate.

#### 5.3.2 Future development directions of chatbots in ophthalmology education

To fully harness the potential of chatbots in ophthalmic education, future efforts should prioritize advancements in technological integration and the establishment of appropriate institutional frameworks. First, incorporating chatbots into VR or AR platforms can offer students immersive clinical training experiences (104), particularly in standardized simulations such as those used in objective structured clinical examinations (OSCEs). Second, the adoption of RAG architectures—in which chatbots are connected to authoritative medical databases and clinical guidelines—can significantly increase the accuracy and professionalism of generated content, thereby mitigating the risk of hallucinations. Third, establishing robust regulatory mechanisms and ethical guidelines tailored to ophthalmic education is essential to ensure the safe, lawful, and pedagogically sound deployment of chatbot technologies. As noted by Sabaner et al. (105), only through the coordinated advancement of both technological capabilities and regulatory policies can AIGC become a truly transformative tool in the modernization of medical education.

### 5.4 Formulating appropriate regulations

While AIGC is being put into pedagogical use, we should improve the laws and regulations to prevent the risks associated with AI misuse and strengthen the regulatory system for AI. Managing the use of AI in ophthalmic education requires strong policies that mandate transparency in the use of AI in research and clinical decision-making and continuously update standards for monitoring AI to create a safe and ethical environment for the use of AI in ophthalmic education (78).

The application of AI in ophthalmology education is developing rapidly worldwide, and different countries and regions have a diversity of technical approaches and regulatory systems. For example, in Europe, the EU passed the General Data Protection Regulation (GDPR) (106) in April 2016, and it officially came into effect on 25 May 2018. The aim of this regulation is to enhance the protection of personal data. Its influence has spread beyond the EU, and the application of AIGC in ophthalmology education worldwide will be inspired by the GDPR. Additionally, the EU AI Act (107) came into effect on 1 August 2024, and it was the world’s first comprehensive regulation for regulating AI. It has strict requirements for AI systems applied in educational and medical scenarios. The EU AI Act and GDPR will improve the quality and safety of ophthalmology education worldwide.

Wills Eye Hospital, a well-known eye research and educational institution in the United States, has been actively exploring the application of VR technology in ophthalmology education in recent years. Carr et al. (108) conducted a study showing that the use of the EyeSi surgical VR system can increase the skill acquisition ability of ophthalmology interns in cataract surgery and reduce errors in surgical operations. These international practices more comprehensively reflect the diverse development of AI technology in different teaching contexts. While adhering to the laws and regulations of each country, absorbing and summarizing advanced international technologies will be more conducive to the application of AIGC in ophthalmology education.

## 6 Conclusion

In the field of ophthalmology, the development and application of AI technology has made great progress, AIGC can generate teaching materials, provide students with simulation training and evaluation and feedback on the teaching effect, but we should also note that AIGC brings many challenges such as misinformation due to the influence of the training dataset, the black box problem, and the infringement of patients’ privacy, etc. In the future, the combination of AIGC, telemedicine, and VR technology will play a greater role in ophthalmology education. In the future, AIGC combined with telemedicine and VR will play a greater role in ophthalmology education. In response to the opportunities and challenges posed by the application of AIGC in ophthalmology education, educational institutions should not only strengthen the regulation of AIGC technologies but also design digital learning curricula aimed at cultivating a new generation of ophthalmic professionals. Through collaboration among researchers, ophthalmology educators, and scientific research institutes, a foundational framework can be established to train medical professionals who are well adapted to the emerging paradigm of AI + medicine.
